# Epstein–Barr virus gene expression in human breast cancer: protagonist or passenger?

**DOI:** 10.1038/sj.bjc.6601027

**Published:** 2003-07-01

**Authors:** S A Xue, I A Lampert, J S Haldane, J E Bridger, B E Griffin

**Affiliations:** 1 Viral Oncology Unit, Division of Medicine, Imperial College Faculty of Medicine at St Mary's, Norfolk Place, London W2 1PG, UK; 2 College of Life Sciences, Shaanxi Normal University, Xian 710062, People's Republic of China; 3 Department of Histopathology, Hammersmith Hospital, Du Cane Road, London W12 ONN, UK; 4 Department of Histopathology, New Cross Hospital, Wolverhampton, UK

**Keywords:** breast carcinoma, EBV gene expression, invasive lymphocytes, oestrogen receptor, p31 EBV DNA

## Abstract

The presence and transcriptional expression of Epstein–Barr virus (EBV)-encoded genes, oestrogen receptor (ER) status and degree of lymphocyte infiltration were evaluated in 15 mastectomy-removed breast cancer samples, mostly of ductal origin. With regard to these parameters, the tumours were heterogeneous. Viral genes, including EBNA1 – a universal EBV marker – and others, selected in part on the basis of expression in other EBV-associated carcinomas and/or presence in an epithelial cell immortalising subfragment p31 of viral DNA, were detected in up to 40% of the breast malignancies. The small viral RNAs, EBERs, were not observed. In culture, p31 EBV DNA, alone among EBV fragments, stimulated the growth of human breast-milk epithelial cells. There was no correlation between viral and ER expression and tumours were heterogeneous with regard to their invasive lymphocytes: of three studied in detail, one contained none, another had (mainly) T-lymphocyte aggregates on the tumour periphery, and a third (BC 12) was infiltrated with both T- and B-lymphocytes. BC 12 differed in several aspects from other malignancies in expressing a transcriptional activator (BZLF1) associated with overcoming virus latency, and failing to express a viral oncogene, BARF1. Arguments are given for EBV as a protagonist cocarcinogen in some breast malignancies.

Breast cancer is well-recognised as a heterogeneous condition and a major cause of death among women. About 800 000 new cases per year are reported worldwide, with one in 11 women estimated to develop this malignancy. In developed countries, >200 cases per 100 000 women per year are seen, 10–15% of these coming from families with a previous history of breast carcinoma (Parkin *et al*, 1999). Breast cancer susceptibility genes, including BRCA 1 and 2 (found on chromosomes-17 and -3), exist, adding support for genetic predisposition for some forms of this malignancy (Masood and Kameh, 2002). Late exposure to a common virus, such as human cytomegalovirus, has been suggested as a risk factor for breast carcinomas (Richardson, 1997), and Yasui *et al* (2001) argue that delayed infection with Epstein–Barr virus (EBV) may also be a risk for this malignancy. For EBV, there is evidence for its presence and expression in subpopulations (up to 50%, but frequently less) of breast tumours (Labrecque *et al*, 1995; Luqmani and Shousha, 1995; Bonnet *et al*, 1999; Joab, 2000; Fina *et al*, 2001; Grinstein *et al*, 2002; Kleer *et al*, 2002; see the discussion by Magrath and Bhatia, 1999). Data that illustrate direct entry of this virus into epithelial cells by routes involving an epithelial-specific receptor (Sixbey *et al*, 1987; Yoshiyama *et al*, 1997) or cell–cell contact with infected lymphocytes (Bayliss and Wolf, 1980; Speck and Longnecker, 2000) have been put forward. In one breast cancer study, the presence of the virus appeared to be associated with a more malignant phenotype of the tumour (Bonnet *et al*, 1999).

In another instance, that of gastric carcinoma, where EBV appears to be associated with a subpopulation (10% or so) of the tumours, virus presence and expression was found only in the epithelial cell population (Galetsky *et al*, 1997). In culture, EBV has been shown to encode genes capable of growth stimulating (immortalising) epithelial populations in tissue explants (Griffin and Karran, 1984; Karran *et al*, 1990) by a mechanism associated with telomere survival and ablation of cell senescence (Gao *et al*, 2002). Based on its well-recognised ability to stimulate cell growth, and the ubiquitous appearance of this virus in the human population, it should perhaps not be surprising to find EBV as a contributing factor to initiation or growth of carcinomas other than those – notably the head and neck tumour of poorly differentiated epithelial cells, nasopharyngeal carcinoma (NPC) – classically associated with it (IARC, 1997).

Whereas with NPC, EBV has been found to be present and expressed in 100% of the tumours, why only a subset of them appear to have a viral association is a question that arises with regard to breast and gastric carcinomas. Arguably, the latter tumours may be more heterogeneous than NPC. Alternatively, failure of detection of the virus may reflect low viral loads in the case of the breast and gastric malignancies, particularly from economically affluent parts of the world, as compared with high viral loads in NPCs, generally from less affluent populations. Further, the role of the virus in malignancy, as exemplified by retention and gene expression, may differ in the one case (NPC) from that in the others. In this study, we were attempting to answer some of these problems as they apply to breast cancer, by readdressing the question of EBV gene expression in a sample of tumours taken by mastectomy from patients in a single British hospital, over a short period of time, thus minimising other factors (environment, diet, viral strains, etc.) that might also contribute to the malignancy. Our data show interesting variations among the tumours, some of which might be harnessed for therapeutic purposes.

## MATERIALS AND METHODS

### Patients

Seventeen tumour biopsy samples were collected, in accordance with and approval of the local ethics committee, from patients presenting with suspected breast malignancy to New Cross Hospital, Wolverhampton, UK, in 1997. Fifteen of these (BCs 1–8, 10–13 and 15–17) were confirmed as breast carcinomas and two, BCs 9 and 14, not confirmed as such but rather identified as a cervical lymphadenopathy and a Hodgkin's disease (HD) tumour, respectively, were used here as controls. Of the breast carcinomas, 12 were ductal in origin, either grades I (BC 3), II (BCs 1, 5, 6, 11, 12 and 17) or III (BCs 7, 8, 10, 13 and 16). Of the others, BC 2 was a poorly differentiated anaplastic lobular cancer (probably grade III), BC 4 (grade III) showed lobular differentiation but with ductal elements, and BC 15 (grade I) was a tubulo-lobular carcinoma. Seven out of 15 of the breast cancers (BCs 3–6, 11, 12 and 15) were oestrogen receptor (ER) positive and the others were negative. The median patient age was 62 years. BC 9, the cervical lymphadenopathy, was designated as ‘possibly of viral origin’, and BC 14, a nodular sclerosing HD, had high numbers of Reed Sternberg cells. For molecular analyses, fresh biopsies were transported on dry ice to the site of analysis, and stored at −70°C. For histochemical analyses, paraffin blocks were kept at −20°C. Other controls used for this study included a North African NPC (C15) passaged as a xenograft in nude mice and containing 30 copies of the EBV genome (Busson *et al*, 1988), a primary African Burkitt's lymphoma (from Xue *et al*, 2002), Asian NPCs (from Hong Kong) and numerous EBV-carrying lymphocyte cell lines.

### Growth stimulus of human epithelial cells *in vitro*

Fragments covering the whole of the EBV genome from the transforming B95-8 strain of virus were transfected in culture into confluent layers of cells derived from human breast milk by a standard protocol (Karran *et al* 1990), and they were left to grow or senesce. In each case, using duplicate cultures, survival of cells over time was visualised by staining with Leishman's reagent. Growth stimulus was obtained with one subgenomic fragment, p31 DNA. As indicated elsewhere (Gao *et al*, 2002), this 40 kbp component of EBV DNA includes among its genes those encoding CST/BART transcripts, which to date have been identified in all EBV-positive tumours (reviewed in Smith, 2001), and an oncogene, BARF1, with functional homology to human colony-stimulating factor (CSF) receptor (Strockbine *et al*, 1998), a product of the proto-oncogene, c-*fms*. Both CST/BARTs and BARF1 are expressed in EBV-positive nasopharyngeal (Hitt *et al*, 1989) and gastric (Zur Hausen *et al*, 2000) carcinomas.

### Expression of EBV genes

**Figure 2:**
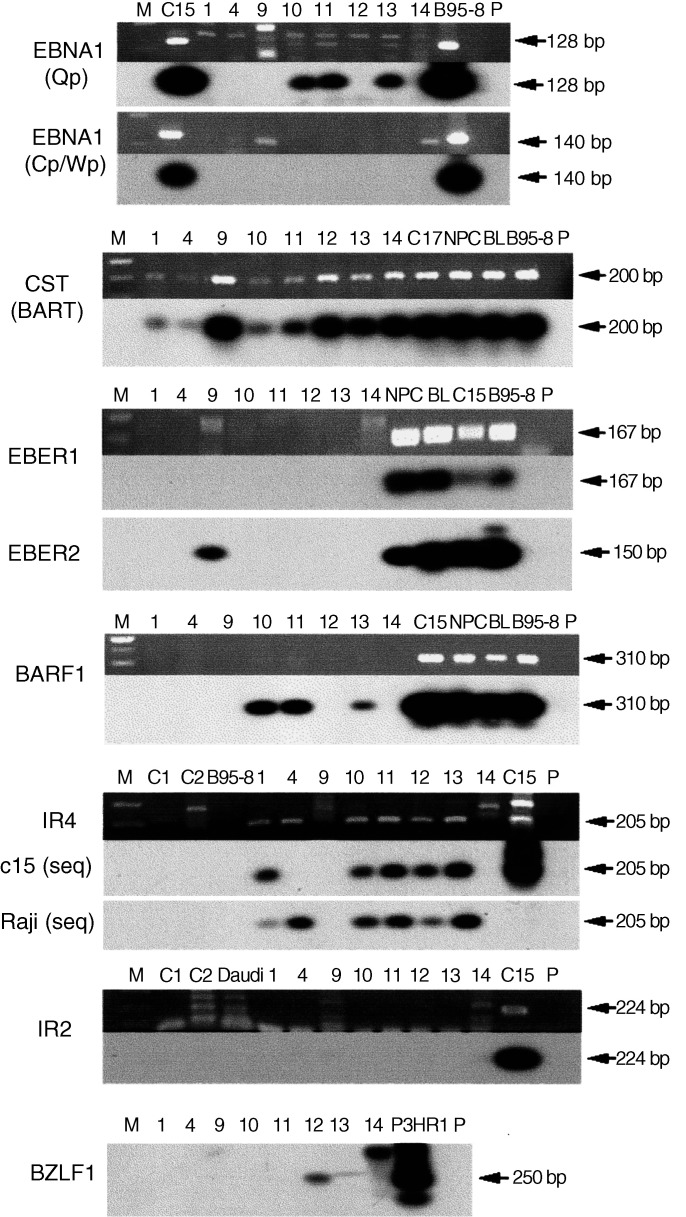
Analysis of expression of EBV RNA in viral DNA-positive breast cancer biopsies (BCs 1, 4, 10–13). EBV genes whose transcriptional expression was monitored include: EBNA1, an essential gene for viral maintenance; three genes (CST/BART, LF3/IR4 and BARF1) expressed in other epithelial cell malignancies (Hitt *et al*, 1989; Xue *et al*, 2000, 2002); small viral RNAs (EBERs 1 and 2) of unknown function but frequently expressed in EBV-associated tumours (Arrand, 2000); BZLF1, an intermediate-early viral lytic cycle marker; and BHLF1/IR2, not universally found in carcinomas but often highly expressed in B-cell lymphomas (Xue *et al*, 2000). Also analysed, but found negative (and not shown), are viral membrane proteins, LMP1 and 2A, associated with growth of B cells, and BHRF1, a viral homologue of *Bcl*-2 (see IARC, 1997). Biopsies, BCs 1, 4, 10–13, are EBV-positive breast cancers, and, except for BC 4, mostly of ductal origin; BC 9 was from a cervical lymphadenopathy, clinically questioned as ‘possibly of viral origin’; and BC 14 from an HD. For the latter two cases, the original designation BC is retained here, although these were subsequently shown not to be breast carcinomas. Other controls include NPCs (both of Asian and North African origin) and a xenograft (C15; see Table 1), a Malawian Burkitt's lymphoma (BL) and B95-8, a prototype strain from a mononucleosis patient. In the EBNA 1 assays, expressions from both latent (Qp) and lytic (Cp/Wp) promoters were analysed. For IR4/LF3 hybridisation, probes corresponding to sequences in C15 and Raji were used (Xue *et al*, 2000). Tracks M contain molecular weight markers.

Viral markers chosen for examination in this study (Figure 2

and legend) include the small EBV RNAs (EBERs 1 and 2) frequently used in searches for EBV (Labrecque *et al*, 1995; Brink *et al*, 2000), the EBV nuclear antigen, EBNA1, responsible for viral maintenance and found expressed in most or all virally associated tumours (IARC, 1997), and a variety of other genes associated with expression in NPC (Hitt *et al*, 1989; Xue *et al*, 2000), or associated with epithelial cell immortalisation in culture (Griffin and Karran, 1984; Karran *et al*, 1990) or, alternatively, thought to be B-cell specific (Xue *et al*, 2000, 2002).

### Isolation and amplification of viral DNA and RNA

RNA was extracted from fresh frozen tumours, or cells, and purified as a pellet, using a standard guanidinium–caesium chloride method, as described (Xue *et al*, 2000). DNA was recovered by dialysing the supernatant from RNA preparations. For analyses by polymerase chain reaction (PCR for DNA) or reverse transcriptase PCR (RT–PCR, for RNA), primers and probes described previously (Xue *et al*, 2002) were generally adopted. For EBER1 and EBER2, the primer pairs used, respectively, were (forward) 5′-AGGACCTACGCTGCCCTAGA and 5′-AAAACATGCGGACCACCAGC, and (reverse) 5′-CCCTA-GTGGTTTCGGACACAC and 5′-GACAAGCCGAATACCCTTCTC. Products were confirmed by using as probes radiolabelled 5′-GTTTTGCTAGGGAGGAGACG (for EBER1) and 5′-GCTCCG-GGGGAGGAGAAGAGAG (for EBER2). For the CST/BARTs, the forward and reverse primers were 5′-TGCGCCTGGAAGTTGTAC-TCCCGGAA and 5′-CTACCGCCACGCGTCAGCAAA, respectively, and the probe was 5′-GTCTTTGACCTGGAGGGCATC. For EBNA 1 expression, both the viral Qp (latent) and Cp/Wp (lytic) promoters were analysed. To avoid possible contamination, nested PCR was not used. After 39 cycles of amplification, PCR products were separated by electrophoresis on 1% agarose gels containing ethidium bromide, and their identities were verified by Southern blot hybridisation. For IR4 hybridisation, probes from both C15 and Raji sequences were used (Xue *et al*, 2000).

### Immunohistochemical evaluation of markers on breast cancer tissues

Antibodies to CD45 (leucocyte common antigen), CD3 (T-cell specific) and CD79a (B-cell specific) (DAKO, Denmark House, Cambridgeshire) were used in tissue examinations. Sections were cut from formalin-fixed, paraffin-embedded blocks and incubated with optimally diluted primary antibody for 60 min, washed (3 ×) with phosphate-buffered saline (PBS), developed using the DAKO Duet/HRP system, and visualised with diaminobenzidine (DAB).

### Excision of epithelial cell populations from tumour tissue

Blocks of frozen tissue from BC 10 and 11, mounted on freezing chucks with OCT (Ames) and sectioned at 4 *μ*m, were placed on glass slides, stained with haematoxylin and eosin (H & E), and microscopically inspected for the presence or absence of lymphocytes. Total RNA was isolated from excised sections devoid of lymphocytes and reanalysed by RT–PCR for expression of two EBV genes, EBNA1 and CST/BARTs (Hitt *et al*, 1989; Smith, 2001).

## RESULTS

### Stimulation of growth of human epithelial cells by p31 EBV DNA

**Figure 1:**
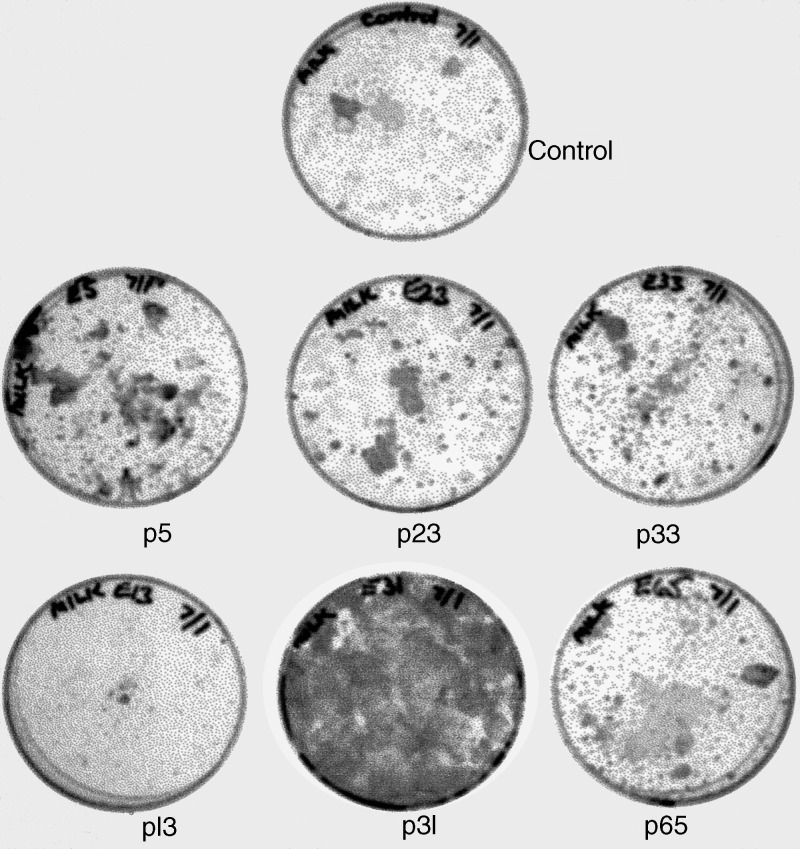
Growth stimulation of human breast-milk cells by subfragments of EBV DNA from a recombinant cosmid library (Griffin and Karran, 1984), made from a transforming strain of the virus, B95-8. One fragment in particular, p31, has the capacity to rescue epithelial cells in primate biopsies from senescence in culture (Griffin and Karran, 1984; Karran *et al*, 1990; Gao *et al*, 2002). Cells from long-term milk cultures were visualised with Leishman's stain. Only p31 DNA, among fragments from this library, was found to have the ability to growth stimulate the human epithelial cells, as shown. In other cases, as well as in control untransfected cultures, only small clusters of dead or dying cells were observed with time.

Others have shown that breast cancer-derived cell lines can be infected with EBV (Speck and Longnecker, 2000). We asked whether a subgenomic fragment of EBV DNA (recombinant cosmid p31; Griffin and Karran, 1984) – which stimulates epithelial cells from populations of young New World primate (marmoset) parotid, kidney and nasopharynx tissues to proliferate indefinitely in culture (Griffin and Karran, 1984; Karran *et al*, 1990; Gao *et al*, 2002) – will likewise stimulate the growth of human breast-milk epithelial cells (Chang *et al*, 1982), and whether this property is confined to the p31 viral subgenomic fragment. Arguably, if EBV has a role in the genesis and/or growth associated with breast cancer, then growth stimulation in these primary cell cultures might result from their transfection by one or more viral fragments. In this experiment, as shown in Figure 1

, only the p31 EBV subfragment was capable of effecting growth stimulation, showing that viral genetic information, which induces survival in other primate epithelial populations, also uniquely stimulates the growth of human milk-derived epithelial cells.

### EBV DNA and gene expression in the breast tumours

Whole-cell DNA taken from the 15 breast carcinoma biopsies, and controls, were examined for the presence of EBV DNA, using as viral marker an early gene, LF3, which contains the repetitive IR4 sequence, and is found expressed in many tumours, including NPCs and Burkitt's lymphomas (BLs) (Xue *et al*, 2000, 2002). The IR4 repeats are present in high copy numbers (ranging from 22 to 33) in different cells and tumours (Xue *et al*, 2003). By PCR, using 39 cycles of amplification and probes and primers described elsewhere (Xue *et al*, 2002), this gene was detected in six out of 15 (40%) of the breast tumours (BCs 1, 4, 10–13) examined (data not shown). All but one (BC 4) of these tumours were of ductal origin, and BC 4 had ductal components. Only 50% of the EBV-positive tumours (BC 4, 11 and 12) carried ERs. The two lobular tumours among our samples and the other ductal tumours, which included the only two Grade I malignancies investigated, were negative.

For exploring transcriptional expression of viral genes in the EBV-positive malignancies, we focused on the CST/BART, LF3/IR4 and BARF1 genes – since these were often found expressed with high frequency in EBV-associated carcinomas (Hitt *et al*, 1989; Xue *et al*, 2000) – and the membrane functions, LMP1 and 2A, expressed in some of these malignancies, as well as the gene for the nuclear antigen, EBNA1, essential for the maintenance of EBV in cells (IARC, 1997), but expressed at low levels. Expression of other viral genes, including the small RNAs (EBERs), often observed in high copy numbers and argued to be diagnostically indicative of EBV (Brink *et al*, 2000) – but not found so in other cases (Takeuchi *et al*, 1997; Sugawara *et al*, 1999) – and BZLF1, BHRF1 and BHLF1/IR2 (see legend to Figure 2), were also explored. None of these gene transcripts (except the EBERs) has been widely used in previous studies investigating EBV in breast cancers, but from recent data on other tumours (IARC, 1997; Sugawara *et al* 1999; Xue *et al*, 2000, 2002; Zur Hausen *et al*, 2000) seemed potentially appropriate for this purpose.

All the EBV-positive breast cancers (BCs 1, 4, 10–13) were found to express the CST/BART and LF3/IR4 transcripts (using protocols that allow for sequence variations, as described in Xue *et al*, 2000), and three out of six expressed BARF1. EBNA1 expression was found in the same three out of six tumours; very low level gene expression, as indicated by work on NPCs (Hitt *et al*, 1989), probably accounts for the fact that EBNA1, and possibly BARF1, was not detected in all the tumours. Notably, no expression of EBERs1 or 2, nor the other viral gene transcripts analysed, LMP1/2A, BHLF1/IR2 and BHRF1, was identified in any of these tumours, nor in BC 14, an EBV-positive HD. EBER2 expression was observed, however, in the cervical lymphadenopathy, BC 9, and expression of other viral genes, such as EBER1 and LMP1, was identified in control cells (not shown), indicating that negative data in the breast cancers did not reflect methodology problems. BZLF1 was found in one malignancy (BC 12), which was infiltrated with both T- and B lymphocytes (see below). Data from some of our studies are summarised in a combined Figure 2, and in Table 1

**Table 1:** EBV gene expression in breast cancer and controls

	**Breast samples**								
**EBV genes**	**BC 1**	**BC 4**	**BC 10**	**BC 11**	**BC 12**	**BC 13**	**9**	**14**	**C 15**
BZLF1	−	−	−	−	+	−	−	−	+
CST (BART)	+	+	+	+	+	+	+	+	+
IR4/LF3 (Raji seq)	+	+	+	+	+	+	−	−	−
IR4/LF3 (C15 seq)	+	–	+	+	+	+	−	−	+
EBNA1 (Qp)	−	−	+	+	−	+	−	−	+
EBER2	−	−	−	−	−	−	+	−	+

Negative in breast cancers: EBER1, EBNA1 (Cp/Wp), BHRF1, IR2/BHLF1, LMP1, LMP2A. BC 1=NHS BSP grade II ductal carcinoma; BC 4=mixed pattern carcinoma, ductal elements present; BC 10=high-grade ductal carcinoma; BC 11=ductal carcinoma; BC 12=NHS BSP grade II infiltrating ductal carcinoma; BC 13=NHS BSP grade III invasive ductal carcinoma; 9=cervical lymph node; 14=HD; C15=NPC xenograft (Busson *et al*, 1988).

.

When viral gene and ER expression data were compared, there was no correlation, ruling out hormonal control over EBV expression in these tumours.

### Analysis of invasive lymphocytes in the breast tumour biopsies

It has been argued by some that invasive, virally infected B lymphocytes might explain the presence of EBV in breast cancers (Brink *et al*, 2000; Chu *et al*, 2001; McCall *et al*, 2001). For this reason, in our molecular analyses (cited above), we largely focused on viral genes associated with EBV-positive carcinomas, rather than those drawn from the literature on B cells (summarised in IARC, 1997). In addition, for reasons of interest and possible relevance, we examined the status of the EBV-positive tumours with regard to invasive lymphocytes. Cell morphology in paraffin-fixed EBV-positive breast cancer sections, BCs 1, 4, 10–13, was examined by H & E staining, with lymphocyte populations detected using the leuckocyte common antibody, CD45. In this study, few if any lymphocytes were observed in the tumour population in BC 10, whereas in BCs 1, 4, 11 and 13 occasional lymphoid aggregates, located mainly on the periphery of the tumours, were observed. An interesting exception was found in BC 12, where heavy lymphoid infiltration was observed in the tumour.

**Figure 3:**
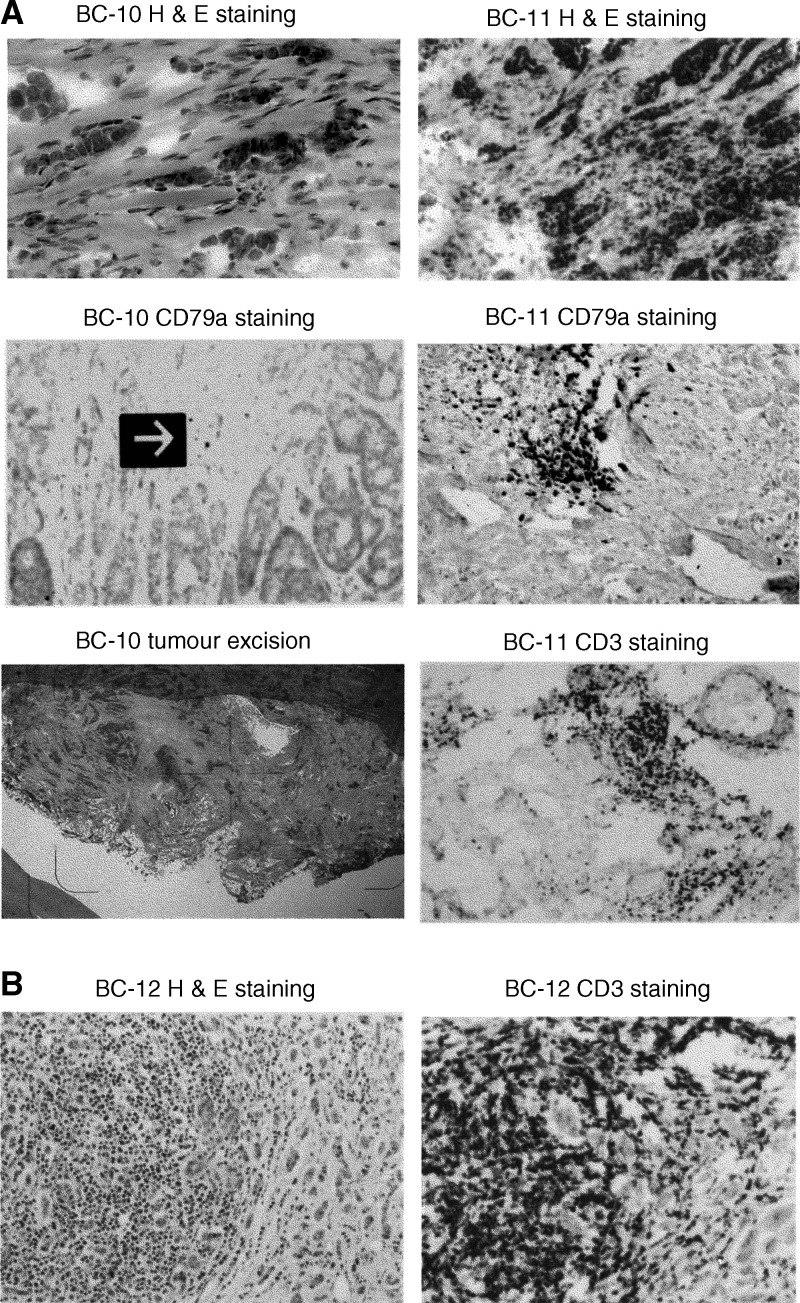
Histology of variants of EBV-positive ductal carcinoma biopsies (BCs 10, 11 and 12). (Magnification × 200). **(A)** Panels (at top) show H & E (dark) staining of paraffin-embedded, frozen sections of BC 10, and BC 11. Section were also stained using antibodies CD79a (for B lymphocytes) and CD3 (for T lymphocytes), as noted. No lymphocyte staining was observed in the tumour population in BC 10, as confirmed by laser dissection microscopy (by Dr B Terris). Any lymphocyte staining in BC 10 was confined to the stroma, as indicated (arrow, middle panel on left), whereas in BC 11, a single patch comprising <5% of the tumour population stained positive with CD 79a (middle panel on right), although most of the lymphocytes within the tumour stained with the T-cell marker (bottom panel on right). Sections free of stroma-containing lymphocytes in BC 10, as illustrated, or freed of B cells in BC 11 by dissection (not shown) were subjected to a separate round of RT–PCR analysis (see Figure 4). (**B**) BC 12, a tumour with inflammatory cells, was stained by H & E (at left), with CD3 (at right) and with CD79a (not shown). This tumour, as shown on the left panel, was heavily infiltrated by lymphocytes, the large majority (about 95%) of which proved on staining to be T cells, as shown (on right). Some CD79a-staining cells within the tumour were observed.

Further, staining studies were carried out on sections to identify the nature of the lymphocytes, using monoclonal antibodies CD79a and CD3 to stain B- and T-lymphocyte populations, respectively. Again, little or no staining was seen in the epithelial cells in BC 10, using either antibody (not shown), although occasional staining cells were observed in the stroma (as indicated in Figure 3A

, middle panel at left). In most of the other tumours, except BC 12, staining showed invasive lymphocyte populations to be mainly of T-cell origin (CD3^+^/CD79a^−^) (not given). In BC 11, one patch of intratumour B cells (CD79a^+^), as shown (Figure 3A, middle panel at right), was observed. BC 12, on the other hand, differed from the other tumours in containing large numbers of lymphocytes (see Figure 3B, H & E staining). Most of these were T cells, as shown in Figure 3B (CD3^+^, at right), although about 5% of them stained with the B-cell (CD79a) marker (not shown).

### EBV gene expression in excised epithelial cells

**Figure 4:**
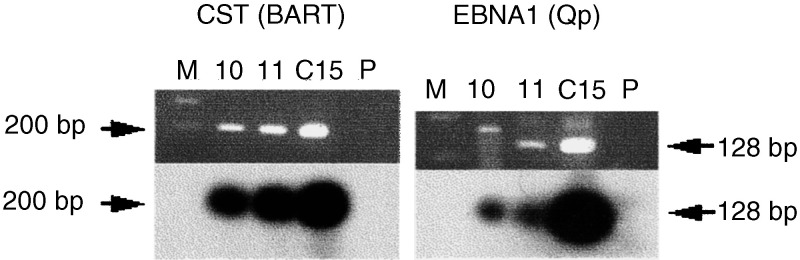
RT–PCR results on BCs 10 and 11, after selection for lymphocyte-deficient epithelial sections (see Figure 3). EBV gene expression (CST/BART and EBNA1, as noted; see also Figure 2) was reinvestigated on RNA isolated from sections with negligible invasive lymphocytes. Primers used for amplification and probes are the same as in Figure 2. RNA from the NPC xenograft C15 (with 30 copies of EBV DNA; Busson *et al*, 1988) was used as positive control and primers only (P) as a negative control. Track (M) contained 100 bp DNA ladders (Promega).

To refute further the arguments that EBV in human breast cancer materials merely reflects the possible presence of EBV-positive B cells in the tumour, we re-examined the status of fresh frozen samples of BC 10 and 11. First, these were subsectioned and stained with the relevant antibodies to B- and T-lymphocytes, as described above. Then, sections where staining showed no or negligible lymphocyte components were excised (as illustrated in Figure 3A, bottom panel at left) and used to make fresh RNA. If invasive lymphocytes had been the source of the EBV gene expression pattern we previously observed (Figure 2), then quantitatively the load should be diminished or abolished by their removal. On re-examining expression of CST/BARTs and EBNA1 in these tumours (Figure 4

), however, the data showed that little or no differences were observed in gene expression between selected samples and total biopsy (Figure 2) samples, arguing for the presence of EBV in the tumour cells themselves.

### Role of amplification in the detection of EBV gene expression

In one PCR study (McCall *et al*, 2001), the importance of amplification cycles was suggested to be of possible significance for the detection of EBV in breast cancers. Our data imply that use of fresh, rather than fixed, sample materials is also a critical factor, as is the choice of viral genes for analysis. We examined viral load in different samples by semiquantitative RT–PCR as a contributing factor in detection, using the CST/BART transcripts observed in all the tumour materials as a marker. For this study, we chose two breast tumours with different levels of expression, BC 1 (weak) and BC 12 (strong) (Figure 2), and the lymphadenopathy (BC 9) and C15 tumour (both very strong) as controls. Using the same primers as elsewhere (Figures 2), and scoring as positive band detection by ethidium bromide staining (as in Figure 2) after 30 cycles of amplification, expression was detectable in controls only; at 35 cycles, BC 12 was positive; at 40 cycles, expression was also observed in BC 1. These data (not given) emphasise the low levels of gene expression in EBV-positive breast tumours in general, and in some in particular, but do not distinguish among high viral loads in a few cells only, or very low levels in many. They confirm the arguments about loads put forward elsewhere (McCall *et al*, 2001).

## DISCUSSION

The herpesvirus EBV, a causative agent for infectious mononucleosis, has been associated with various malignancies with frequencies that vary from 100% (in poorly differentiated NPC) to a few percent only (in adenocarcinomas) (IARC, 1997), or, as recently discussed, up to 45% (nine out of 20) in fibroadenomas from HIV-positive individuals (Kleer *et al*, 2002). In some studies, EBV has been identified as a component of breast cancers (Labrecque *et al*, 1995; Luqmani and Shousha, 1995; Bonnet *et al*, 1999; Fina *et al*, 2001; Grinstein *et al*, 2002; Kleer *et al*, 2002) in up to 50% of cases examined (Bonnet *et al*, 1999). Interestingly, whereas association of EBV with about 10% of human gastric carcinomas (Shibata and Weiss, 1992; Rowlands *et al*, 1993; Galetsky *et al*, 1997; Takano *et al*, 1999; Yoshiyama *et al*, 1997; Zur Hausen *et al*, 2000) is accepted as real and meaningful, the association of this virus with human breast cancer remains controversial in spite of the well-documented positive findings, and higher frequencies (reviewed by Magrath and Bhatia, 1999).

Here we have explored the hypothesis that in studies where EBV has not been identified in breast cancers, either in terms of its genome or expressed genes, experimental design – which may fail to allow for low viral loads, or rely on the expression of EBV gene patterns more appropriately associated with B-cell malignancies – may be a key factor (Brink *et al*, 2000; Chu *et al*, 1998; McCall *et al*, 2001). Further, the use of fixed archival samples by some investigators (Gaffey *et al*, 1993; Horiuchi *et al*, 1994; Lespagnard *et al*, 1995; Chu *et al*, 1998, 2001; Glaser *et al*, 1998), rather than fresh materials, can compound the problem as recovery of nucleic acids from such materials may be hampered, and fixation can result in DNA (mutational) damage (Williams *et al*, 1999). From the data, it is not clear, moreover, that the small viral RNAs (EBERs) need be good markers for EBV in this malignancy (Bonnet *et al*, 1999), and several studies have relied heavily on them to define the presence or absence of EBV in breast samples (Horiuchi *et al*, 1994; Glaser *et al*, 1998; Brink *et al*, 2000). Moreover, a further factor may account for some discrepancy in that different histological variants of this malignancy may vary in their association with EBV. It is notable and of possible relevance that in two studies on medullary carcinomas, which share many characteristics of lymphoepithelomas and among the breast cancers have a generally better prognosis, EBV was not identified (Gaffey *et al* 1993; Lespagnard *et al* 1995). However, these data were not consistent with that reported elsewhere (Luqmani and Shousha, 1995). Perhaps, more than anything else, the data on EBV in breast cancer argue strongly for a larger scale and more systematic study of this topic than, with one possible exception (Fina *et al*, 2001), has been carried out to date.

In our current studies, we have used well-defined fresh biopsy materials, and focused mainly on viral genes that appear from other work (Griffin and Karran, 1984; Hitt *et al*, 1989; Karran *et al*, 1990; Zur Hausen *et al*, 2000; Gao *et al*, 2002) to have relevance for epithelial cells. We initiated our work by identifying the ability of one subfragment of EBV DNA (p31), but not others, to stimulate the growth of human milk epithelial cells in culture (Figure 1). Then, using two viral genes (CST/BARTs and BARF1) found within p31 DNA, another (LF3) from the same region of the genome that has been found expressed in all EBV-associated tumours where it has been explored to date (Xue *et al*, 2000; 2003), and the EBNA1 gene required for maintenance of EBV, and others, we made a detailed investigation of a sample of British breast cancer patients. The samples were fresh and all came from patients seen in a single hospital within a short period of time. All were treated by mastectomy, and most were of Caucasian origin with ductal carcinomas. In all, 40% (six out of 15) of these were found to carry the EBV genome and express viral genes. With four viral genes (CST/BARTs and LF3/IR4 (six out of six positive), and EBNA1 and BARF1 (three out of six), the data were incontrovertible – although one would predict that EBNA1 at least is probably expressed in all these tumours, but below the level of our detection methods. As in some other cases of carcinoma (Takeuchi *et al*, 1997; Sugawara *et al*, 1999; reviewed by Arrand, 2000), the small viral RNAs, EBERs, which provide good markers for analysing EBV association in some other tumours, proved not equally useful for the breast samples. In some cases, particularly for example that of the tumour BC 10, with no lymphocyte infiltrates, other viral genes but not EBERs were expressed. Our data on viral gene expression are shown in part in Figures 2 and 4 and summarised in Table 1. Data on lymphocyte infiltration into the tumour populations are given in Figure 3. We found that in the positive breast samples from a British population, even in fresh biopsies, EBV expression levels were low as compared, for example, with those found in Asian NPCs or African BLs, and required optimisation of techniques for detection.

In general, the EBV-positive cases were younger (median age 54 years) than their EBV-negative (median age 63 years) counterparts. In particular, we draw attention to the case of patient BC 12 (aged 33 years), whose tumour was unique among those examined, both with regard to its viral gene expression pattern and its intense chronic inflammatory infiltrate. Adjacent normal breast lobules also showed a similar inflammatory infiltrate, clinically suggestive of an enhanced host response. Alone among the breast tumours studied, BC 12 expressed a transcription product associated with the active spliced version (Figure 2) of one of the intermediate-early viral genes, BZLF1 (or ZEBRA), that disrupts EBV latency (Miller, 1990). In the HD, BC 14, studied as a control, a product possibly from an unspliced transcript of this gene was expressed. Although it has been suggested that only T lymphocytes invade epithelial cells (Hayday *et al*, 2001), and our histology studies showed that any B cells associated with the breast tumour populations under investigation lay mainly at their periphery, uniquely in BC 12 many infiltrates were observed in the tumour. Although these were mainly T lymphocytes (Figure 3B), there were B cells among them. Thus, the BZLF1 expression detected in this particular tumour could have been a product of the invasive lymphocytes, which in turn provoked an enhanced host response in the patient. Arguably, as shown recently (Speck and Longnecker, 2000), EBV-infected lymphocytes may be uniquely capable of targeting breast epithelial cells. Assuming tumours such as BC 12 to make up a subclass of breast cancers, it would be of therapeutic relevance to explore whether harbouring a potentially oncolytic version of EBV can be put to therapeutic use, as discussed for other viruses (Chiocca, 2002). Of possible relevance to this suggestion, introduction of the BZLF1 (or a similar EBV gene) *via* an adenoviral vector was shown to inhibit the growth of an NPC xenograft in a mouse model (Feng *et al*, 2002). Harnessing endogenous EBV gene expression for therapeutically useful purposes may thus be worthy of further exploration.

From accumulating data in the literature (see above) and those presented here, we argue that the relevant question(s) now is not *whether* EBV can be present and persist in breast carcinomas, but rather *what* its role might be. The notion of this virus as a ‘harmless’ passenger' (Labrecque *et al*, 1995) seems implausible in view of its association with many malignancies of different cell types, and the ability of its genes to contribute to cellular growth (immortalisation) in culture. In a recent comprehensive study of EBV, it was declared to be ‘*carcinogenic to humans*’ (IARC, 1997). Bonnet *et al* (1999) showed that the more aggressive breast tumours in their studies were to be found among the EBV-positive cases. Our data were mainly derived from grade II/III breast malignancies that were evenly split with regard to their EBV status, whereas neither of the grade I malignancies available for study proved to be EBV positive. One of the functions that EBV can confer upon epithelial cells is to extend their life span, as illustrated in Figure 1 and elsewhere. In other studies, primary epithelial cells immortalised by a subfragment of EBV appeared to lose their viral information (classically called ‘hit and run’ immortalisation), yet did not go through crisis; throughout their growth cycle their telomeres remained constant in length (Gao *et al*, 2002). In recent studies on gastric carcinoma cell lines (Kassis *et al*, 2002), the presence of EBV was found to increase cell motility and mobility. The data overall argue for EBV as a protagonist with regard to some carcinomas, including breast cancer, possibly acting as a ‘carcinogenic agent’ that can maintain telomerase activity *in vivo*. The virus seems unlikely to be a mere passenger.

In the newer molecular approaches, transcription profiles and microarray techniques are being used to modify taxonomies for breast cancer. For example, Perou *et al* (2000), using 65 different surgically removed samples identified great variations in the patterns of gene expression, with sets of genes (clusters) contributing to independent patterns. The largest cluster included expression of genes associated with cellular proliferation. Oestrogen receptors were also detected in a cluster. Microarray technology offers great potential for unravelling some of the problems associated with breast cancer. In future, it would seem relevant to adapt it to include EBV gene expression.
